# Foreign Summary

**Published:** 1839-06-22

**Authors:** 


### FOREIGN SUMMARY.

*Clinical Lecture on Hepatitis.* By ROBERT CARSWELL, M. D.—Gentlemen: I have selected for the chief subject of lecture to-day three cases of hepatitis, which have been under treatment for a considerable time. This disease presents itself to our consideration in several important points of view. Although of less frequent occurrence than inflammation of some other parenchymatous organs, and, in this climate, comparatively seldom proceeding to the same extent as the latter, still it is a disease which the physician is frequently called upon to treat under circumstances of considerable difficulty, both as regards the obstinacy of the disease itself, as well as its sometimes complicated character, the effects to which it gives rise, and the manner in which it terminates.

With regard to the comparative frequency of inflammation of the liver, I may observe that it most certainly occurs much less frequently than inflammation of some other parenchymatous organs, as the lungs, for example; and this statement would be corroborated by the numerical appreciation of any given number of cases of hepatitis and pneumonia, occurring in a given time, or in a given number of patients; and, whatever the comparative result might be, still there would be an additional number in frequency above the numerical quantity in favour of the frequency of pneumonia over that of hepatitis. I mean to say that the numerical method, even, would not altogether enable us to determine the comparative frequency of the two diseases, because the means which we possess of establishing our diagnosis of the presence of inflammation in the liver and lungs respectively, are not equally certain in both. The physical signs of pneumonia, whether the disease exists alone, or is combined with bronchitis or pleuritis, are, in general, so positive, that we can seldom fail in detecting its existence. But the physical and physiological signs of hepatitis are not only few in number, but mostly of a negative kind, inasmuch as they may accompany not only other morbid conditions of the liver, but also have their source in neighbouring organs or tissues. Hence, as I have said, the probability of reckoning among cases of hepatitis diseases of a different nature. Of these diseases the most frequent are duodenitis, gastro-duodenitis, colonitis, and peritonitis, occupying chiefly the region of the liver. In fact, hepatitis is, as I have already said, frequently complicated with these diseases, and must partake of the symptoms which belong to them.

However, as illustrative of the cases which I have to bring under your notice, I may state here the symptoms which are generally considered as indicating the existence of acute hepatitis, and which are divided into two groups, viz., those of a *general* and those of a *local* character. The *general* symptoms are those of fever, either of an inflammatory character, at the commencement and during the progress of the disease, or they may, at an early period, or towards the termination of the disease, present the adynamic or typhoid character. But this latter form of fever is, I believe, rarely, if ever, observed in this country, unless when the hepatic disease is complicated with gastritis or gastro-enteritis, and is certainly the worst form of complication which the disease can assume. The fever is accompanied by a rather strong and full pulse; thirst; generally a yellow-furred tongue; sickness, or vomiting of a bilious fluid, of a yellowish or greenish colour; or it may be of a *brownish* colour; and this latter appearance is, perhaps, always indicative of inflammatory congestion of, and hæmorrhage from, the gastro, or gastro-intestinal mucous membrane.

And here I must digress, for a moment, from the point immediately before us, to remark, that the vomiting of this brown fluid matter (which consists of blood that has been effused, and deprived of its red colour by the chemical action of the gastric juice,) may not always depend on *inflammatory* congestion of the mucous membrane of the stomach, or duodenum. It may be the result of *mechanical* congestion of this membrane, in consequence of obstructed circulation in the liver, whereby the return of the blood through the vena porta is more or less impeded; and, indeed, I have seen this congested state of the liver acting mechanically, and so obstructing the portal circulation as to give rise to severe and extensive hæmorrhage from the stomach and intestines. When, therefore, the brown or black vomiting accompanies hepatitis, we may regard either as a sign of inordinate inflammatory congestion of the liver, or of the gastro-intestinal mucous membrane, followed by hæmorrhage.

To the other general symptoms which I have mentioned may be added a hot and dry skin; scanty and high-coloured urine; generally a sallow complexion. Jaundice is not necessarily an attendant on hepatitis; nor do I believe that our researches yet enable us to explain why it is present in some cases and absent in others. And the same remark applies to the biliary secretion, which, in acute hepatitis, is sometimes deficient or suppressed, at others apparently little modified in quantity or quality; and hence the frequently unsatisfactory curative indications derivable from the state of the alvine evacuations in this disease; or which may be stated thus:—the *absence* of bile in the evacuations is merely a sign of its defective secretion, but not of the pathological change on which it depends; and, on the other hand, the *presence* of bile in the evacuations is not a certain sign that extensive disease may not exist in the organ by which it is secreted.

Such are the general symptoms most commonly observed at the commencement of an attack of acute hepatitis, and when not complicated with a similar affection of the stomach and intestines, or respiratory organs. With regard to the local symptoms of the disease, the most important of them are pain, tenderness on pressure, and swelling in the region of the liver.

Of these symptoms pain is by far the most important, whether the sensation be felt by the patient, independently of any external cause, or be occasioned by pressure, position, or otherwise. Whatever may be the degree of the pain felt by the patient, it is always aggravated by pressure, a circumstance which serves to distinguish it from hepatalgia, which, if not relieved, is not increased by this means. It is, also, generally increased by the respiratory movements, by the erect position, and by decubitus on the left side. It may be confined to the anterior, lateral, or posterior portions of the right hypochondrium; to the right or left lobe, or both. In some cases it is entirely confined to the region of the liver; in others it radiates through the back, and more frequently upwards, to the right and left shoulders.

Some physicians attach great importance to this seat of the pain, and consider it as even pathognomonic of hepatitis; whilst others regard it as not only of little importance when present, but instance numerous cases in which it has not occurred at all. In this latter statement I fully concur, as I have seen undoubted cases of hepatitis in which the pain in the right shoulder was absent. Why there should exist this diversity in regard to this symptom has not been satisfactorily ascertained; but it is more than probable that it arises in a difference in the seat of the inflammation. This circumstance certainly influences materially the *degree* of the pain felt in hepatitis; for in this disease, as in inflammation of all parenchymatous organs, pain may be entirely absent if the inflammation is slight or comparatively trifling, although the inflammation may be severe, if confined to the central portion of the organ; and, on the contrary, whatever may be the degree of the inflammation, if seated *superficially*, it is always accompanied by some degree of pain, felt or excited, and is always of the acutest kind in this situation.

And it appears to be equally certain that the increase of pain, in certain positions of the body, as when the patient lies on the left side, is owing to the same circumstance, and especially when the peritoneal surface of the liver is implicated in the disease.

The next local syptom of hepatitis, and which, when taken in conjunction with pain, is of importance, is enlargement or tumefaction of the liver. This alteration sometimes occurs to a considerable extent, so as to be easily recognised by the touch or percussion. It is, of course, most readily perceived when it affects the anterior portion of either lobe, when it protrudes beneath the false ribs, or into the epigastrium. These regions of the body then appear fuller, communicate the dull sound of the liver on percussion; and, when the right lobe is much enlarged, the false ribs are carried forwards, or even tilted upwards. There do occur, however, cases of hepatitis in which this enlargement of the right lobe is not so easily detected, or cannot easily be distinguished from pneumonia of the inferior lobe of the right lung, or a pleuritic effusion of the same side, but as neither of our patients presented this complication, I shall not discuss this part of the subject.

Before proceeding to the description of the cases which I have to bring before you, I may briefly allude to the terminations of acute hepatitis. The most frequent of the terminations of acute hepatitis is into that of the chronic form of the disease, and which is much more frequently met with than the former, although I may observe that there are few diseases of the liver which have not been included under the denomination of chronic hepatitis, and few cases of the disease of long standing which are not complicated with chronic gastro-duodenitis.

The most favourable mode of termination, however, is that of *resolution*, as it is called, and is indicated by the diminution and the gradual disappearance of the general and local symptoms which I have enumerated, viz., of the fever and gastric symptoms, the pain and tenderness, and tumefaction of the liver. Much importance has, indeed, and justly, been attached to the disappearance of these local symptoms, as indicating the progress of the disease towards a favourable termination. However, in chronic forms of the disease, the tumefaction and tension which accompanied the acute stage may have subsided or entirely disappeared, while some degree of pain may still remain, excited either by pressure or in particular positions of the body, as is exemplified by the case of one of our patients.

By far the least frequent mode in which acute hepatitis terminates, is in that of suppuration or abscess; and, in the great majority of cases, the occurrence of this event is announced most frequently, and first of all, by *hectic fever;* that is, by periodical rigors of greater or less severity, followed by febrile excitement, a rapid and weak pulse, frequent, and sometimes profuse perspirations, general pallor, anorexia, and prostration. Besides these general symptoms, as indicating the occurrence of suppuration, and the most important of which are the rigors and hectic fever, are observed certain states of the local symptoms. The tumefaction of the liver does not subside in proportion as the pain diminishes; or, the pain remaining severe, or even increasing, is accompanied by an increase of the tumefaction, by the formation of a circumscribed swelling, in which fluctuation is detected by percussion. The formation of abscess may, however, occur without our having any sign of its existence, as when it is deep-seated, or occupies the inferior or superior surface of the liver; or it may be situated in the anterior parietes of the organ without pointing forwards, rendering it difficult or impossible to detect it by the presence of fluctuation. In such cases, however, it may sometimes be detected by the presence of a doughy or boggy sensation, as it is called, communicated to the touch,—a sensation which acquires great diagnostic value by the existence of rigors and hectic fever at the same time.

I shall not occupy your time by any farther general observations on this last stage of the disease, nor on the various modes employed by nature and art to afford a salutary exit to the contents of the hepatic abscess. In one only of our patients are there grounds for believing that the inflammation has terminated in the formation of abscess. The subject of this case is Ellen Cox, ætat. 37, admitted 7th November, and has, consequently, been more than three months under treatment in the hospital. This patient is described as being of a sanguineous temperament, with a sallow complexion; unmarried; subject to very hard work, and exposed to cold and wet, the injurious effects of which were increased by the kitchen in which she worked being cold, and having a stone floor. Under the influence of these exciting causes she had, at the age of 23, an attack of rheumatism, which lasted for a period of six or seven months, and since then has been subject, during the winter, to pain in the right side, accompanied by dry cough. A year and a half ago she had jaundice, with the usual symptoms of hepatitis, for which she was treated antiphlogistically. She has since, occasionally, had pain in the right side; and a fortnight before her admission, having taken cold, from her feet having been wet, the pain in the side was much aggravated, the cough and difficulty of breathing also much increased. On examination she presented the following symptoms:—Pain in the right hypochondrium, increased by pressure, and extending to the shoulders; cannot lie on left side; sickness; pain in the epigastrium; bowels relaxed, and motions pale coloured; tongue red, and furred at the back; respiration hurried and difficult, with sonorous râle over *left* side; viscid, mucous expectoration; pulse 95; skin hot, dry, and harsh, with a slight tinge of yellow; no yellowness of conjunctiva; headach; sleeps badly; catamenia very irregular, sometimes not appearing for twelve months.

From these symptoms you will readily perceive that this is a well-marked case of acute hepatitis, and, what adds considerably to its gravity, is its occurrence after a previous attack, from which the patient had not completely recovered. You will also readily perceive that it is a case of hepatitis complicated with some degree of gastro-enteritis, the presence of which was marked by the red and furred tonngue, pain at the epigastrium, and sickness, and the relaxed state of the bowels, and that notwithstanding the deficient state of the biliary secretion. But, besides this gastric complication, there was also another, which increased still more the gravity of the principal affections, viz., bronchitis; and this you will observe, was on the left side of the chest, and which renders it more than probable that it was a concurrent affection, produced by the same exciting cause which gave rise to the hepatitis, rather than a complication of this disease from transmission of the inflammation by contiguity or otherwise, as frequently happens when bronchitis, or, it may be, pneumonia or pleurisy, occurs on the right side of the chest, a larger extent of which is contiguous to the inflamed liver. With this concurrent affection and the gastro-enteric complication, together with the previous bad state of health of the patient, and, above all, the already diseased condition of the liver, we can feel but little surprise that this case has proved so intractable under the most active means of treatment. On the day of her admission she was bled to twelve ounces; the following day to the same amount; two days after to twelve ounces more; once again to the same amount, after the same interval of time; and on the succeeding day she was ordered to be bled ad deliquium, when the loss of eighteen ounces was followed by fainting. By means of this active antiphlogistic treatment, together with the administration of five grains of blue pill, from three to four times a day, both the general and local symptoms were relieved. The condition of the liver, however, and more especially the pain felt in the region of this organ, rendered it necessary to have recourse to the local detraction of blood; fifteen leeches were therefore applied; and four days after were repeated, to the number of twelve. Notwithstanding these means, and although the mouth had for some time been affected by mercury, she complained of darting pain in the region of the liver. She was again bled, but only six ounces, the pulse being 84, but weak; and on the following day was bled to the same amount, with some relief.

From this time up to the 18th of December, nearly three weeks, the pain varied considerably in degree, as well as the other symptoms; the pulse then rose to 100, when blood was again taken from the arm, to the amount of six ounces, and was repeated two days afterwards; and again, for the last time, on the 1st of January. The mercury was laid aside after the gums became affected, and was afterwards resumed and carried to the same extent, but was ultimately omitted, not only in consequence of the disease making no progress towards a cure, but from the supervention of symptoms indicating the commencement of suppuration. This occurred on the 31st of December, more than two months after the commencement of the acute symptoms of hepatitis. The patient then complained of rigors, and a throbbing pain in the right side; the rigors have since occurred daily, with considerable perspiration; and the character, as well as the degree of the pain, has varied much, having been partially relieved by poulticing, warm fomentations, opiate liniments, and, on two or three occasions, by leeching.

Although we have repeatedly examined the region of the liver, and, in particular, that part of it to which the pain is chiefly referred, we cannot discover the sensation of fluctuation; but there is distinctly felt a puffiness, or doughyness, of a rather circumscribed extent of the anterior surface of the right lobe, and below the margin of the false ribs. It is in this situation that the pain is still complained of, and is much increased even by slight pressure. Under these circumstances, and the continuance of the rigors and hectic, there can hardly be a doubt of the existence of an abscess; but I do not think that it would be advisable to have recourse to surgical means either to obtain relief to the patient, or increase her chance of recovery, until more positive evidence is obtained as to the existence and situation of the abscess. The treatment of the case is altogether palliative; the strength of the patient is supported, as it has been all along, by such food as the stomach will retain or digest, chiefly beef-tea, milk, and eggs, for the gastric complication has rendered any other means inadmissible. Sickness and vomiting have frequently occurred during the course of the treatment, and which have been relieved or arrested by the use sometimes of creosote, at others by the hydrocyanic acid. The bronchial affection, too, although it has yielded to the general antiphlogistic treatment, still exists, but on what condition of the lungs it depends, I am unable to say, as I have avoided examining the chest, in consequence of the fatigue and suffering it would occasion the patient. I need hardly observe that opiates have been employed during the course of the disease to procure some relief from suffering, and repose.

I ought to have stated that blisters have been applied to the region of the liver; on one occasion to the chest, for the relief of the bronchial affection; and also to the abdomen, owing to the occurrence of acute pain in that region.

I have dwelt the more on this case because it is a good example of acute hepatitis, occurring under the influence of the ordinary exciting causes; because it presents, in succession, the more usual symptoms of the disease, and one, at least, of the most common of its complications; because of its probable termination in suppuration; and, lastly, because of its exemplifying, in a striking manner, the difficulty of speedily and effectually curing this disease, especially in persons who have already been affected with it.

The second case is one of a much less complicated character, but has not on that account yielded readily to the active measures which have been employed. The attack was the *fourth* from which the patient, a female, 26 years of age, named Sarah Monk, had suffered within a period of six years. The first attack lasted two months; she had then been treated with mercury. It is not stated whether, in the two subsequent attacks, she employed any treatment. The last attack occurred at Christmas last, and had not left her since free from pain.

On the admission of the patient, the 2d of November, she complained of a sharp, shooting pain in the right hypochondrium, increased by pressure, deep inspiration, and cough, extending to the back and right shoulder; she can lie on the right side only; skin hot and dry, of a yellowish tinge; respiration natural; tongue clean and moist; pulse full and firm; a preternatural sound accompanies the contraction of the ventricles.

In this case the principal symptoms of acute hepatitis are present and well marked, although not of great severity; and it may be said to be free, or nearly free, from all complication. Considered in this point of view only, a comparatively speedy and favourable result might have been anticipated. Such, however, has not been the case, for the patient has been more than three months under treatment, and is not altogether free from pain in the region of the liver.

From the patient's own statement, she recovered from her first attack at the end of two months, under the use of mercury, and apparently without the aid of antiphlogistic means. The subsequent attack was of longer duration; and the obstinacy of the present one is, in all probability, to be attributed, in some degree at least, to the previously diseased condition of the liver, as in the preceding case. In this respect, therefore, this case resembles the previous one, in which the patient had suffered several attacks before that for which she was admitted into the hospital; and hence the prognosis was equally unfavourable in both, being, however, greatly aggravated in the former, in consequence of its most important complications, viz. the gastro-enteritis and bronchitis.

I have said that the present case might be regarded as being without any marked complication. The preternatural sound heard in the region of the heart could not be looked upon in this light. This sound persists, little, if at all, modified by the treatment which has been employed for the hepatic disease; and from the circumstance of its long continuance, in the absence of any obvious cause, is, in all probability, owing to disease of the valves, and, from the situation in which the sound is heard, the aortic valves.

The treatment employed in this case was actively antiphlogistic. General bloodletting, however, was exclusively employed; that is, without local bleeding by leeches or cupping. During the first fortnight forty-seven ounces of blood were taken at five different bleedings, the last bleeding having produced fainting. Considerable benefit followed this treatment, with which was combined the internal administration of mercury, but which, it may be observed, neither up to a late period, nor subsequently, could be made to affect the mouth, except in the slightest possible degree.

After the amelioration to which I have alluded there was a relapse, indicated by an accelerated pulse, and increase of tenderness in the hypochondrium, which was combated by a small general bleeding and the application of a blister to the region of the liver, which, after having been repeated several times, was followed by considerable relief from the local pain.

Since this period, the commencement of January, recovery has gone on progressively but slowly. She now complains of no pain when in bed, very little while up, but it is still produced by pressure. As the disease has for some time consisted of little more than a certain degree of pain, without any perceptible enlargement of the liver, and as the secretion of bile is still very scanty, this patient is taking slight alterative doses of the blue pill, in combination with Dover's powder and henbane, at night, and the extract of taraxacum, with the tinctura humuli and the spiritus ætheris nitrosi, during the day; together with the occasional use of blisters over the situation of the liver in which the pain is produced by pressure.

The third and last case which I have to bring under your notice is that of Sarah Pipe, ætat. thirty-five, who was under treatment for a period of six weeks, and who left the hospital a few days ago convalescent.

The following were the symptoms presented by this patient on her admission:—Great pain in the epigastrium and upper part of the umbilical region, increased by the slightest pressure; nausea, and frequent vomiting; colicky pains, and great constipation: great thirst; tongue brown and red at the point; severe headach; skin dry and hot, of a slightly yellow tinge; pulse 108, and hard; urine small in quantity and high-coloured.

From these symptoms you will not, certainly, recognise the presence of hepatitis. They are, on the contrary, the symptoms of gastro and entero-peritonitis, the gastro-peritonitis being the more marked of the two complications. Besides these symptoms, however, there was also felt by the patient, at her admission, and when she lay on her left side, a *dragging* pain in the right hypochondrium. It was, in fact, chiefly from the presence of this symptom that the existence of hepatitis could at all be admitted. Indeed, I bring forward this case with the view that you may contrast it with the two former cases, in which the hepatic disease predominated. In the present case the affection of the liver, which must have been slight, appeared to be secondary to, or a complication of, the gastro-peritonitic disease.

The pain in the epigastric and umbilical regions, increased by the slightest pressure, indicate already the peritonitic character of the inflammation; and the obstinate constipation which existed, not only at the commencement of the attack, but for a considerable time afterwards, and in spite of the most active purgatives, is an equally marked character of peritoneal inflammation. For constipation, of an obstinate kind, is by no means a symptom of *enteric* inflammation: I mean of inflammation of the mucous membrane of the intestines. When this symptom is present in enteritis, it is ordinarily at the commencement, and soon terminates in an opposite state, viz., diarrhœa, which did not occur in this case.

The brown tongue, red at the point, and the great thirst, were the chief symptoms referrible to gastritis, or gastro-enteritis. It must, however, be remembered that they are by no means pathognomonic symptoms in these affections of the digestive mucous membrane, and may accompany peritonitis alone. The state of the pulse did not afford us any assistance in establishing our diagnosis in this case.

The treatment consisted, at first, in the use of terebinthinate enemata, and full doses of castor oil, which operated but sparingly, and which increased the sickness and vomiting. These symptoms recurred for several days, and were either mitigated or subdued by the use of creosote. The constipation was only overcome by large doses of croton oil, as much as eight minims having, at one time, been required to move the bowels; and this is the more remarkable that blood-letting was employed at the same time, and to the extent of producing fainting on two or three occasions. Mercury was also employed with the view of affecting the mouth; and blisters were applied twice to the abdomen. It was not until the mouth became sore, about ten or twelve days after her admission, that decided and permanent relief from pain was obtained, and that the bowels acted by means of mild cathartics. The pain afterwards complained of was chiefly, if not exclusively, confined to the epigastric region, diminished gradually but slowly, by the application of leeches and blisters; and the strength and appetite of the patient began to return, at first under the use of the nitric acid, and afterwards of the sulphate of cinchonine.

I shall terminate this case by remarking that the gastric affection, which, at the commencement, was in some degree obscured by the peritonitic, became more marked, and, as it were, isolated, after the subsidence of the latter. The persistence of the pain in the epigastric region only when pressure was applied to the part, or occurring after taking food, with defective appetite, were the chief, if not the only symptoms which the patient presented during the last four weeks she remained in the hospital; and in proportion as the disease assumed the chronic form, the patient was benefitted by the tonic treatment which was ultimately adopted.—*Lancet.*

---

*Three Cases of Abortion, with Symptoms of Poisoning, produced by the use of Rue.* By H. Th. Helie.—Case I. A young girl of very short stature, but of robust constitution, who had had a very difficult labour at the age of sixteen, brought on a miscarriage in the third or fourth month of pregnancy, in the following manner, as she confessed:—She sliced three fresh roots of rue, as big as her finger, and, boiled them in a pound and a half of water down to three cupfuls, which she took in the evening at a draught. She was immediately seized with a dreadful pain in her stomach, which was soon followed by so great and general a disorder that she thought she was going to die; she saw every thing through a cloud, tottered, and felt giddy, and, as it were, intoxicated. Soon afterwards there were violent and continual efforts to vomit, but she only brought up a little blood. This state lasted the whole night. On the following day the symptoms diminished, and at the same time she began to experience colic, which was slight at first and more severe afterwards, the fits being separated by long intervals. Towards the evening of the second day they became violent, and followed one another in quick succession. There was now a small discharge of blood, then large clots were thrown off, and abortion took place with ease in a few minutes, forty-eight hours after the decoction of rue had been swallowed, the girl not having been confined to her bed. The symptoms caused by the rue went off in a few days.

Case II. Maria ——, aged 25, a servant in town, had been in the country with a farmer for five days, to get well of an indisposition with which, she said, she had been recently attacked. During the first two days she went out, and had a good appetite. On the third day she was suddenly seized with continual vomiting, which was difficult and painful. There was considerable fever and thirst, and she drank a large quantity of service cider* and wine-and-water, which she immediately vomited up. The vomiting had now lasted two days, accompanied by great weakness, twisting movements of the limbs and trunk, rotatory movements of the head, and delirium, or rather reverie, when M. Hélie saw the patient, on the 5th Dec. 1835. She was then in a sleepy state, from which she was easily roused; she answered questions well, but with slowness and some difficulty; the eyes were injected, the face somewhat coloured, and without expression: one might have said that she was drunk. Her sight was dim, the pupil contracted, some fever, with a large and soft pulse, and but little heat of skin. The urine had been suppressed since the vomiting had begun, and there were no alvine evacuations. The tongue was somewhat red just at the edges; the epigastrium was slightly painful. On feeling the abdomen, Dr. Hélie perceived that the patient was about seven months pregnant. The pertinacity of the girl in denying her pregnancy, and this sudden attack of vomiting, accompanied by unusual symptoms, led Dr. Hélie to suspect the employment of some drug to procure abortion; but his inquiries were for the time fruitless. There was nothing to announce the beginning of labour; the abdomen was quite yielding, and there was no discharge. On the other hand, her general plumpness and good complexion forbade the supposition that there was some recent disease. He therefore confined himself to prohibiting the hurtful drinks which the patient had been using for the last two days, and to prescribing a decoction of barley, with low diet, &c.

The vomiting was soon allayed, but all the other symptoms continued. On the morning of the 6th she seemed to suffer still more. At eleven o'clock, her face, her general state, and all the symptoms, were the same as on the previous day, but of diminished intensity. On raising the bed clothes, M. Hélie was struck with the characteristic odour of parturition; and between her thighs there were two children, still attached to the placenta, which had also been thrown off. They seemed to have reached six and a half or seven months of fœtal existence, but one was much larger than the other. They were both dead, but there was nothing to show that they had lived after their birth, nor was there any trace of external violence. There was a considerable quanity of blood and water in the bed; the uterus was well contracted. The pains, according to the girl's account, had begun the day before, and delivery had taken place an hour preceding this visit; and though it had been very painful at the moment, several persons present in the room had not the least suspicion of it. This day and the next produced but little change in the symptoms. There was still a state of somnolence and stupor, reverie, movements and twisting of the limbs, and frequent moans; stools and urine were passed, and the lochia flowed.

---

* A drink is made in some parts of France from the fruit of the service-tree.—*Translator's Note.*

She took roast meat in wine, according to country usage.

On the evening of the 8th there was swelling of the breasts, with fever, delirium, and convulsive movements of the limbs, which were both violent and continual: until this period Maria had not been very ill. In consequence of this exacerbation she fell into a state of extreme debility; the vomiting returned, the matter brought up consisting of green bile, and of food and drink, which were thrown up as soon as swallowed.

On the 9th, the symptoms increased, and there was distension of the abdomen. On the morning of the 10th, there was extreme weakness, prostration, somnolence, stupor, an intoxicated expression of countenance, and frequent moaning, with the articulation of a few monosyllables; there was also reverie, with subdelirium, and there had been for the last two days enormous swelling of the tongue, which was red, but covered with a thick whitish coat. The pupil was constantly contracted, the eyes dull, the vision confused, the pulse feeble, soft, very small, and beating only thirty times in the minute, but regular; the temperature of the skin was below the healthy standard. The arms were effected with a twisting motion, and the head rolled to the right and left. The epigastrium was tender on pressure, but the rest of the abdomen was supple and not painful. The uterus had returned to its natural situation. There were continual bilious vomitings; the patient refused to eat or drink, and had grown sensibly thinner; the lochia were suppressed. These symptoms, unaccompanied by peritonitis, strengthened Dr. Hélie's belief that abortion had been excited by some narcotico-acrid substance. In fact, he learned that Maria had taken a decoction of rue leaves, without being able to ascertain in what dose or how many days she had employed it.

On the two following days, the 11th and 12th, the state of the patient was nearly the same. Two large blisters had been applied to the thighs, and cataplasms to the epigastrium; barley-water had been prescribed for drink; and, on account of her extreme weakness, a few spoonfuls of light broth, which was at first vomited, but afterwards retained; two blisters were then applied to the legs, and cold sugar and water was given for drink.

There was afterwards some mitigation of the symptoms.

On the 13th there was a remarkable change: the symptoms of poisoning gradually went off, and were changed into those of typhus fever, the disease not becoming less serious.

On the following days, a considerable improvement took place; the tongue returned to its natural state; the salivation stopped; the convulsion of the limbs ceased; the understanding seemed less obtuse; the pulse remained equally weak and slow, but the skin was no longer cold. There was an attack of fever every evening, and vomiting when the paroxysm came on, but at no other time. The urine continued to pass involuntarily, but no longer accumulated in the bladder. The lochia had returned. She was ordered to take milk as her sole aliment, and gum-water with milk. The improvement continued; the typhoid state went off in a few days; and the evening febrile attacks did not return. The abdomen yielded to pressure, and was not tender; but there was obstinate constipation for some days which was overcome by two ounces of manna. The urine now ceased to flow involuntarily; the face regained its natural expression, and the understanding its powers. The slowness of the pulse was one of the symptoms which lasted the longest.

On the 30th of December, Maria returned to service, and some days afterwards was able to work again.

Case III.—A young girl, being four or five months pregnant, and wishing to induce abortion, took for several days a large dose of the expressed juice of fresh rue leaves. She experienced symptoms quite resembling those of the preceding case, and was in great danger. When she was at the worst, among other symptoms were observed prostration, somnolence, excessive general debility, frequent fainting, extreme smallness, weakness, and slowness of the pulse, extraordinary coldness of the skin, and continual non-convulsive movements of the limbs, particularly the arms. As in the preceding case, there came on active inflammation, and considerable swelling of the tongue, with copious salivation. For several days abortion was seen to be coming on, but the fœtus was not expelled till towards the sixth day, dating from the beginning of the symptoms of poisoning. When abortion had taken place, all the symptoms grew milder, and no inflammation of the uterus followed. The symptoms of poisoning, however, lasted at least twelve days, and went off gradually. The girl recovered slowly.—*London Med. Gazette*, *from Annales d'Hygiène.*

---

*On the division of the Sterno-cleido-mastoid Muscle for the Cure of Wry-neck.* By Professor Dieffenbach.—The great success of the operation lately introduced by Stromeyer for the cure of club-foot, has directed the attention of the profession to the benefit arising from the use of the knife in all cases of permanent contraction of the muscles. Professor Dieffenbach gives, in the paper before us, the outline of thirty-seven cases in which he divided the sterno-cleido-mastoid muscle, in one of which only the operation was unsuccessful.

The operation is thus performed. The patient being seated on a chair, one assistant draws the head towards the healthy side, whilst another depresses the shoulder of the affected side. The contracted muscle is in this manner brought to stand further out, and is seized between the thumb and forefinger of the left hand, and drawn powerfully downwards. A strongly-curved bistoury is now introduced behind the muscle, and pushed forward till its point is felt beneath the skin on the other side of the muscle. The edge of the knife is then turned towards the muscle, and its fibres divided by withdrawing it, taking care not to injure the integuments. When the muscle of the left side is that which is to be divided, the knife is introduced in the triangular space formed by the two portions of the muscle about two inches above their insertion, and from this point first the anterior portion, and then, if necessary, the posterior portion is divided. When the contracted muscle is that of the right side, the knife is introduced between the trachea and muscle; the anterior portion is first divided, and then, from a second puncture between the two portions of the muscle, the posterior portion is separated. In the instant of withdrawing the knife the thumb is pressed firmly upon the wound, to prevent the extravasation of blood beneath the skin, and a compress is applied, which is retained in situ by strips of adhesive plaster and a bandage. Two cloths are passed round the neck, with a view to give support to the head, which is allowed to retain its wry position, partly in order to prevent the extravasation of blood, and partly to favour the reunion of the muscle.

In general the wound heals in a few days. There is generally some swelling over the part where the muscle was divided, and fluctuation is occasionally felt, owing to the extravasation of a small quantity of blood. In such a case the compress is applied more firmly, and stimulating embrocations are had recourse to. Sometimes, though very seldom, suppuration takes place; this accident calls merely for the evacuation of the pus, and the simple treatment of the wound.

In some of his first cases, Professor Dieffenbach employed various means of extension, to bring back the head to its natural position; but he afterwards found a stiff collar of pasteboard, so constructed as, from the uneasiness it produced, to force the patient to turn the head in the contrary direction, quite sufficient to restore the natural position.

We shall give one or two of the cases, taken at random, as illustrations.

*Case* 1.—C. Meir, aged twenty-four, affected with congenital contraction of the sterno-mastoid muscle, producing strongly marked scoliosis. When thirteen years of age he began to wear a mechanical apparatus, which he continued for some years, but afterwards gave up, as the scoliosis continued to increase. Both insertions of the muscle were divided, and a compress and bandage applied as already described. Neither extravasation nor suppuration took place. The patient remained in bed ten days; gentle extension of the neck was then employed, and in three weeks the cure was complete, and the position of the head natural.

*Case* 15.—Carl Von Schuck, a very lively boy, had been treated according to the most approved system of orthopedy for a considerable time, without benefit. The muscle was divided; some days after the operation, fluctuation from extravasated blood was perceptible, but augmented pressure produced its absorption. At the end of six weeks the boy left Berlin perfectly straight.

*Case* 37.—A boy aged twelve. The right sterno-mastoid was very much contracted, and the head in consequence approximated to the shoulder. This patient had already been operated upon according to the older method; the muscle had been laid bare and afterwards divided, but the contraction returned as soon as the wound healed. The muscle was again divided according to the same method, and although means of extension were had recourse to during the cure, the contraction again returned. According to the report of the father of the patient, this treatment occupied three months. Owing to the induration and adhesions produced by the two former operations, Professor Dieffenbach found it necessary to divide the muscle near its middle. The after-treatment was as usual; no means of extension were used, simply the pasteboard collar. No extravasation or suppuration followed; the patient was able to go out five days after the operation, and the head regained its natural position.

Of the thirty-nine cases, nine were owing to contraction of the left, and thirty to contraction of the right sterno-mastoid muscle.—*Brit. and For. Med. Rev.*, *from Medicinische Zeitung.*

---

*On the temperature of the Vagina and Uterus before and during Menstruation, and of the Vagina during Pregnancy.* By J. C. G. Fricke, Hamburg.—This paper contains an account of thirty-four experiments made on twenty-four women. The thermometer (Reaumur's) used in examining the vagina was bent at a right angle, and introduced as deep as possible into the vagina, the labia being closed on it; that for the uterus had a very fine and longish bulb, which was introduced from four lines to half an inch into the uterus. The speculum employed was previously warmed. The experiments were made between 10 and 11, A. M. The following conclusions are deducible from the whole observations:—1. That the temperature of the external air affects the axilla, but not the internal parts. 2. That the vagina is always warmer than the axilla and uterus; but that the uterus is warmer than the axilla. 3. That both menstruation and pregnancy have little or no effect on the temperature of the vagina.—*Ibid*, *from Zeitschrift*, *f. d. g. H.*

---

*Belladonna in Erysipelas.*—At a late meeting of the Medical Society of London, the members were occupied with a discussion on the treatment of erysipelas.

Mr. Headland made some remarks on the great variety of treatment recommended by various teachers in this disease, a fact which he thought an opprobrium to the science of medicine. He had read some cases of erysipelas in the Lancet, in which Mr. Liston had employed the extract of belladonna, in small doses, with great effect. He (Mr. Headland) had since tried this remedy in three cases, and the effects were most satisfactory,—more satisfactory, indeed, than those from any other remedies which he had ever employed. From the good effect of belladonna in scarlatina, as well as in the disease in question, he believed that it possessed a specific action upon the skin.—*Lancet.*

